# Routine Histopreparations After Tonsillectomy, Tonsillotomy, Adenotomy or Conchotomy: A Necessary Diagnosis in Times of Dwindling Resources?

**DOI:** 10.3390/jcm15031195

**Published:** 2026-02-03

**Authors:** Givi Magradze, Felix Deffner, Manuel Christoph Ketterer, Christoph Becker, Andreas Knopf

**Affiliations:** Department of Otorhinolaryngology, Medical Center–University of Freiburg, Faculty of Medicine, University of Freiburg, 79106 Freiburg, Germany

**Keywords:** routine histopathology, tonsillectomy, adenoidectomy, neoplasms, health economics

## Abstract

**Objective:** The primary objective of this study is to investigate the prevalence of unexpected findings requiring treatment after routine histological examinations following tonsillectomy, tonsillotomy, adenotomy, or conchotomy (TTAC) in a retrospective study and to discuss whether routine histological examination is useful in patients without clearly defined risk factors or whether it would be better to reduce unnecessary costs and resource utilisation. **Materials and methods**: The present retrospective study encompasses 5709 patients who underwent routine histological examinations following TTAC and were treated as inpatients at the University Medical Center Freiburg, Department of Otolaryngology, Head and Neck Surgery, between 2011 and 2021. The data was collected based on patient characteristics, including date of birth, gender, age of patients at the time of surgery, date of surgery, indication for surgery, tissue examined, and histological result. **Results:** Of a total of 6687 patients who underwent TTAC, 5709 with routine histological examinations were included in the analysis, of whom only four showed abnormal findings, corresponding to an overall prevalence of 0.07%. Three of these four patients were adults. These included two cases of granulomatous inflammation, one instance of Burkitt lymphoma, and one instance of chronic lymphocytic leukaemia/small cell B-lymphoma. Following the exclusion of tuberculosis and sarcoidosis, and the lymphoma board’s decision to adopt a watch-and-wait approach in the case of chronic lymphocytic leukaemia/small cell B-cell lymphoma, only n = 1/0.0175% of patients were found to require treatment. **Conclusions:** The study demonstrated that only four abnormal histological findings occurred in 5709 inpatient TTACs, of which only one, namely Burkitt lymphoma, ultimately required treatment. Consequently, it can be concluded that routine histological examinations following TTAC are not beneficial in patients without clearly defined risk factors, such as blood in the saliva, history of smoking or alcohol consumption, unexplained pain, previous cancer, mucosal changes, or tissue asymmetries. However, in instances where clinical or anamnestic suspicion of malignancy is present, a histological examination should be conducted.

## 1. Introduction

Tonsillotomy, tonsillectomy with or without adenotomy, and conchotomy (TTAC) are among the most common surgical procedures worldwide [1]. In the period between 2011 and 2021, a total of 1,674,248 conchotomies, 756,269 tonsillotomies and tonsillectomies without adenotomy, 195,622 tonsillotomies and tonsillectomies with adenotomy, and 366,100 adenotomies without tonsillotomies or tonsillectomies were performed on inpatients in Germany [2]. The vast majority of patients undergo TTAC for functional or infectious indications, rather than as part of a diagnostic approach to suspected malignancy.

Usually, all tissue material obtained intraoperatively that is initially unremarkable is sent to the pathology department for routine histological examination. In such cases, a histopathological examination is performed using routine staining techniques. If deemed necessary by the pathologist, further immunohistochemical procedures may be conducted. The resources involved in histological processing include the following steps: sample processing in the operating room; documentation of histology using a request form; transportation of the sample to the pathology department for histological examination; and final evaluation of results by the ENT clinic.

It is important to note that all of these procedures are both time-consuming and costly [3]. In the context of mounting financial pressures and personnel shortages within the healthcare sector in Germany, it is imperative to optimise the utilisation of resources [4,5]. This necessitates an evaluation of the diagnostic efficacy of routine histological examination of the aforementioned anatomical regions, and whether it imposes a disproportionate strain on limited resources.

The necessity of routine pathology can be called into question on account of the benign nature of most routine samples from tonsils, adenoids, and inferior turbinates. Consequently, it has been posited that the associated financial outlay and administrative burden exceed the advantages offered by pathology [6,7,8,9,10,11].

However, malignancy was also diagnosed in routine histological examinations of patients with no clinical or historical suspicion of cancer. A systematic review of the literature was conducted to investigate the prevalence of unexpected malignancies in routine histological examinations of tonsil and to discuss the indication of routine histological examination in tonsillectomy samples from patients without clearly defined risk factors. The review identified 37 articles involving a total of 72,322 patients. However, only 11 patients (0.015%) were diagnosed with an unexpected malignancy after routine histological examination following tonsillectomy [11].

At the same time, the incidence of head and neck carcinomas, particularly HPV-associated oropharyngeal squamous cell carcinoma, has increased markedly over recent decades, predominantly affecting adult patients [12,13,14].

The primary aim of this retrospective observational study was to evaluate the prevalence of unexpected histopathological findings requiring treatment after routine histological examination following TTAC under real-world clinical conditions. A secondary objective was to assess the associated resource utilization and to discuss whether routine histopathology is justified in patients without clearly defined risk factors, with particular emphasis on the adult patient population.

## 2. Materials and Methods

The study was conducted in accordance with the Declaration of Helsinki (1975), as revised in 2013, and the study protocol was approved prior to data collection by the local institutional review board (Ethics Committee of the Albert-Ludwigs-University of Freiburg, Approval No. EK-Freiburg 22-1031-retro, 22 February 2022). The study was also registered in the German Clinical Trials Register (DRKS00032486, Date of registration: 8 February 2024). It is also registered in the Freiburg Clinical Trials Register (FRKS004838, Date of registration: 14 August 2023). This study was designed as a retrospective observational analysis.

This retrospective study includes 6687 patients with histological examinations after TTAC who were treated as inpatients at the Department of Otorhinolaryngology, Head and Neck Surgery at the Freiburg University Medical Center between 2011 and 2021. These data originate from the hospital information system “MeDoc” and were collected using Microsoft Excel. Subsequently, patient characteristics such as date of birth, gender, age of the patient at the time of surgery, date of surgery, tissue examined and histological findings were systematically recorded by retrospective review of patient records.

Inclusion criteria are routine histological examinations following inpatient TTAC. Moreover, inclusion criteria include TTAC performed as an inpatient at the Department of Otorhinolaryngology at the Freiburg University Medical Center between 2011 and 2021, with a therapeutic goal (e.g., abscess tonsillectomy). The absence of clinical or anamnestic evidence of specific inflammatory diseases or of malignancy is hereby noted. The histopathological examination findings are to be completed and made available. Furthermore, it is imperative that all relevant findings are available in the hospital information system “Medoc”.

Patients were excluded from the study if the histological examinations after TTAC were performed due to a tumour disease or a suspected tumour disease. Such exclusions included, for example, preoperative clinical or anamnestic suspicion of malignancy and preoperative already confirmed malignancy. Furthermore, patients with incomplete or missing histopathological examination findings were excluded from the study.

The surgical coding was used to identify all patients who underwent at least one of the following procedures between 2011 and 2021: tonsillectomy, tonsillotomy (OPS: 5-281.x; 5-281.y; 5-281.0 - 5; 5-282.x; 5-282.y; 5-282.0 - 1;), adenotomy (OPS: 5-285.x; 5-285.y; 5-285.0 - 1;) or conchotomy inferior (OPS: 5-215.1) was performed on an inpatient basis. The above-mentioned patient characteristics were recorded. The most important inclusion criteria are routine histologic examinations after inpatient TTAC. In addition, all relevant findings must be available in the hospital information system “Medoc”.

Prior to statistical analysis, several consultations were conducted with two members of the Institute for Medical Biometry and Statistics (IMBI), Freiburg. During these consultations, potential analytical approaches were discussed, including subgroup comparisons (e.g., pediatric versus adult patients, diagnostic categories, and tissue types), the identification of potential threshold values for increased occurrence of abnormal findings, and the possibility of defining statistical measures to express the clinical usefulness of routine histopathological examination.

Furthermore, the calculation of binomial confidence intervals was considered. Based on the very small number of abnormal findings, the biostatistical experts recommended an exploratory descriptive statistical approach. Meaningful comparisons between subgroups and the calculation of predictive models were not feasible, as at least 40 abnormal cases would be required to obtain a robust predictor. It was concluded that binomial confidence interval estimation would provide limited additional value given the low event rate.

Statistical analysis was therefore limited to descriptive statistics and performed using IBM SPSS Statistics version 29. Results are presented graphically and descriptively.

## 3. Results

**Figure 1:**
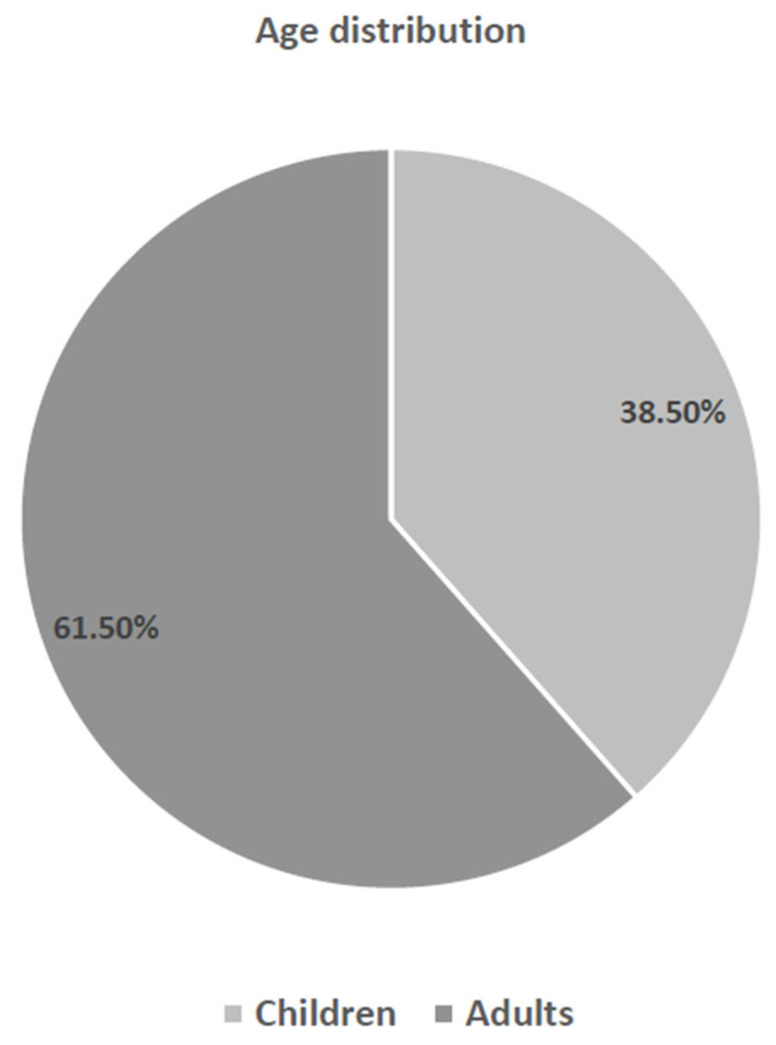
Representation of the percentage breakdown of age distribution.

**Figure 2:**
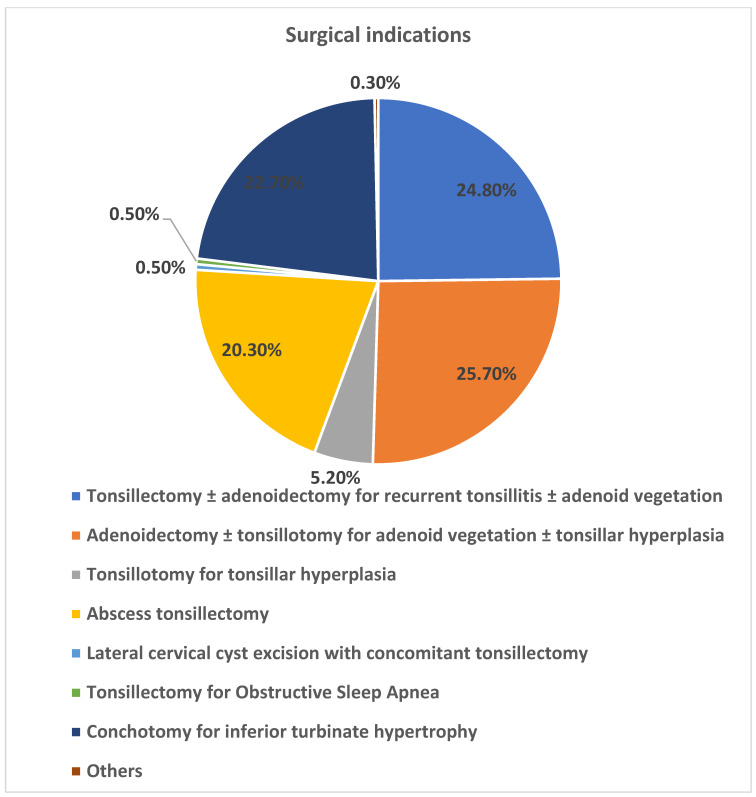
Graphical representation of the percentage distribution of surgical indications.

A total of 6687 inpatient cases with histological examinations after TTAC were initially considered. Following the application of inclusion and exclusion criteria, 5709 patients were included in the study, with a higher proportion of male patients (n = 3407/59.7%). The mean age of the patients at the time of surgery was 24.07 years (range < 1 to 93 years). Of these, 2198 were children and 3511 were adults, corresponding to a percentage distribution of 38.5% versus 61.5% (Figure 1). The following clinical indications resulted in the 5709 surgical procedures: The prevalence of recurrent acute tonsillitis with and without adenoid vegetation (n = 1415/24.8%), adenoid vegetation with and without tonsil hyperplasia (n = 1463/25.7%), tonsil hyperplasia (n = 299/5.2%), and peritonsillar/retropharyngeal/parapharyngeal abscesses (PTA) (n = 1157/20.0%) was documented, lateral neck cyst with tonsillectomy (n = 30/0.5%), obstructive sleep apnea with tonsillar hyperplasia (n = 31/0.5%), nasal concha hyperplasia (n = 1295/22.7%), others (n = 19/0.33%) (Figure 2). A total of 2895 tonsillectomies and tonsillotomies (50.7%), 1048 adenotomies (18.4%), 454 combined adenotomies and tonsillectomies/tonsillotomies (8%), 1293 conchotomies (22.6%), 13 combined adenotomies and conchotomies (0.2%), and 6 combined tonsillotomies/tonsillectomies and conchotomies (0.1%) were examined histologically. A total of 5709 inpatient TTACs were subjected to routine histological examinations, which revealed a total of n = 4/0.07% abnormal findings. Two of these were granulomatous inflammations, one Burkitt lymphoma and one small cell B-lymphocytic lymphoma.

All four patients were male. The presence of granulomatous inflammation was detected during tonsillotomy for the treatment of tonsillar hyperplasia (patient aged 11 years) and during septoplasty with conchotomy for the treatment of nasal concha hyperplasia and septal deviation (patient aged 63 years). During the course of an abscess tonsillectomy, Burkitt lymphoma (in a patient aged 58 years) and small cell B-lymphocytic lymphoma (in a patient aged 69 years) were detected. In both cases of granulomatous inflammation, systemic inflammatory diseases such as tuberculosis and sarcoidosis were excluded after extensive diagnostic procedures. These procedures included chest X-rays, the Quantiferon test, microbiological examinations, immunological laboratory tests, lung function tests, neck ultrasounds, and bronchoscopies with ultrasound-guided lymph node and mucosal biopsies. In both cases of lymphoma, only one, namely Burkitt lymphoma, was treatable. The second patient diagnosed with small cell B-lymphoma did not require treatment following comprehensive diagnostic procedures conducted in the oncology clinic, due to the absence of symptoms, as discussed in the interdisciplinary tumour board. The patient was referred to a practising oncologist for ongoing monitoring. The results demonstrate that of the 5709 inpatient TTACs included in the study, only four exhibited abnormal histological findings. It is noteworthy that among these findings, a mere 1/0.0175%, namely Burkitt lymphoma, ultimately necessitated treatment.

The findings of random inspections demonstrated that, within our clinic, personnel with varying degrees of education (surgical nurses, transport personnel, medical staff) allocated approximately 15 min per case to this routine histological evaluation. This includes sample processing in the operating room, documentation of histology using a request form, transportation of the sample to the pathology department for histological examination, and final evaluation of results by the ENT clinic. It is important to note that the time required for histological examination in the pathology department was not included in this calculation. In our clinic, routine histological examinations are performed approximately 577 times per year after inpatient TTAC, corresponding to a time expenditure of 8655 min (144.25 h) per year.

## 4. Discussion

Routine histopathological examination after TTAC is commonly performed in daily clinical practice. However, our study revealed that clinically relevant histopathological findings in patients without preoperative suspicion of malignancy are exceedingly rare. Routine histological examinations revealed four notable findings. Two of these were granulomatous inflammation, one was Burkitt lymphoma, and one was small cell B-lymphocytic lymphoma. It is noteworthy that all four patients were male. One of the granulomatous inflammations was detected during a tonsillotomy, with the others being identified during a conchotomy. The Burkitt lymphoma and the small cell B-lymphocytic lymphoma were detected during an abscess tonsillectomy. In both instances of granulomatous inflammation, tuberculosis and sarcoidosis were excluded following further diagnostic procedures. In this particular instance of small cell B-lymphocytic lymphoma, the lymphoma board opted for a “watch and wait” approach, given the absence of a perceived necessity for treatment. It has been observed that patients diagnosed with small cell B-lymphocytic lymphoma appear to demonstrate a reduced risk of disease progression following a five-year period. The average risk of progression necessitating treatment is estimated to be in the range of 1–2% per year [15,16]. On the other hand, it seems that Burkitt lymphoma is a very serious diagnosis. It is described in many publications that Burkitt lymphoma is a highly aggressive B-cell lymphoma and the fastest-growing cancer in humans, with a dramatic clinical presentation characterized by rapid growth of the lymphoma and spread to extranodal anatomical sites [17,18,19,20].

The results of the study indicate that, of the 5709 inpatient TTACs included in the study, only four abnormal histological findings occurred. Importantly, three of these four findings occurred in adult patients, whereas only one pediatric patient was diagnosed with suspected granulomatous inflammation. Following the exclusion of tuberculosis and sarcoidosis, and in consideration of the lymphoma board’s decision to adopt a “watch and wait” approach for cases of small cell B-lymphocytic lymphoma, only one out of the four cases (0.0175%) was found to necessitate treatment. This indicates that a total of 5709 routine histological examinations were conducted, identifying only one finding that required treatment. In the present cohort, 3511 patients (61.5%) were adults, while 2198 patients (38.5%) were children. Importantly, clinically relevant histopathological findings were observed predominantly in adult patients, underscoring that the main clinical relevance of routine histological examination lies in the adult population. It is important to note that patients with unilateral peritonsillar abscesses often exhibit clinical features such as tonsillar asymmetry or palatal arch protrusion [21,22], so that at the time of diagnosis of a peritonsillar abscess, it is not possible to assess whether the tonsils were asymmetrical prior to abscess formation, unless there is a history of suspected malignancy or a conspicuous finding on a previous examination. Our study included patients who underwent abscess tonsillectomy for therapeutic purposes and without prior suspicion of malignancy. No squamous cell carcinoma was detected in the routine histological examination of the included patients after abscess tonsillectomy. Unfortunately, as described above, two lymphomas were diagnosed, but one of them did not require treatment, further underscoring the rarity of clinically relevant findings in this setting.

The findings of our study are closely aligned with those of related research, including a systematic literature review from Denmark (2014). This Danish review examined 37 articles containing data on a total of 72,322 patients, investigating the prevalence of unexpected tonsil malignancies in routinely removed tissue in both children and adults. A total of 11 cases (0.015%) of unexpected malignancy were identified. The authors concluded that the extremely low rate of such findings does not justify routine histological examination [11]. A further retrospective review of the PubMed database from 2007, encompassing 20 studies, reported on the results of histological examinations following tonsillectomy, tonsillotomy and adenotomy in 54,901 patients. Six patients (0.011% of the total cases) were found to have unexpected findings requiring treatment. The authors concluded that, given the low incidence of such findings, discontinuing routine histological examination in the United States would result in annual cost savings of approximately $35,467,080 [23]. Two retrospective studies involving a total of over 2000 inferior turbinates, conducted by the Cleveland Clinic and Thomas Jefferson University in the US, showed no findings requiring treatment [9,24].

However, as demonstrated by our study and the aforementioned studies, routine histological examinations of patients after TTAC, in cases where there was no preoperative suspicion of cancer, reveal malignant disease, albeit in very small numbers. Of particular concern is the rising incidence of head and neck carcinomas, particularly oropharyngeal cancer, in recent decades [25,26]. This rise predominantly affects adult patients, reinforcing the need for heightened clinical vigilance in this population. Consequently, in instances where clinical and anamnestic suspicion of malignancy is present, it is of paramount importance that a histological examination is conducted, particularly in patients with a history of smoking or alcohol consumption. Indicators that may be of specific significance in this regard include the presence of blood in the saliva, as well as the presence of B symptoms, which may be defined as weight loss, night sweats, or a history of previous cancer. Further symptoms of concern include persistent pain, a foreign body sensation in the throat lasting for a period exceeding three weeks, odynophagia, dysphagia, otalgia accompanied by normal otoscopic findings, and recurrent epistaxis. Clinically relevant findings include macroscopic abnormalities observed during surgery, such as mucosal changes, tissue asymmetries, palpable changes or cervical masses, which are clear warning signs [23,27,28,29,30]. In such scenarios, targeted histopathological examination is clearly justified and should not be omitted.

A further significant consideration is that routine histological examinations after TTAC are both time-consuming and potentially costly for both ENT clinics and pathology departments. As demonstrated by our results, approximately 15 min per case are required for routine histological processing within our clinic, involving multiple workflow steps and professional groups. When extrapolated to routine clinical practice, this corresponds to an annual time expenditure of approximately 144.25 working hours. Such cumulative time investments represent a relevant burden on available personnel resources. Although a precise monetary cost calculation was not performed, as personnel costs vary substantially between professional groups, institutional structures, and healthcare systems, the reported time expenditure should be interpreted as a surrogate marker of resource utilization rather than a universally transferable economic estimate. In view of the escalating cost pressures and staff shortages that are prevalent within the healthcare sector in Germany, it is imperative to optimise the utilisation of resources. The financial implications of a solitary histopathological examination are such as to impose a considerable economic burden on our clinic and the healthcare system as a whole [3,4,5]. The high workload, particularly in circumstances where there is a shortage of staff, results in extended working hours. These extended working hours are a significant stress factor, which has been demonstrated to increase the risk of health problems and to have a particularly negative impact on the mental health of hospital staff [31,32,33,34]. Mental health issues have been demonstrated to have a detrimental effect on concentration levels, professional performance, and the quality of work produced by hospital staff, thus increasing the likelihood of medical errors being made [35]. A limitation of this study is that immunohistochemical analyses and pathogen-specific testing were not performed systematically, reflecting routine diagnostic practice rather than targeted infectious screening.

In this context, the question must be posed: does a single finding requiring treatment among 5709 cases justify such a high level of human and financial resources? One potential future direction for further exploration is the implementation of a selective approach. Consequently, the indication for a histological examination is contingent upon the presence of a clinically or anamnestically substantiated suspicion. Potential criteria for inclusion may encompass, but are not limited to, the following: conspicuous tissue asymmetries, mucosal alterations, ambiguous pain presentations, a history of cancer, prolonged tobacco use, or alcohol consumption. Adopting a selective approach may enhance efficiency within the healthcare system while maintaining a high standard of patient safety, particularly when applied with careful clinical judgment.

## 5. Conclusions

Consequently, it can be concluded that routine histological examination following TTAC is not necessary for patients without clear risk factors and may in fact be counterproductive. Should a clinical sign or medical history give rise to the suspicion of cancer, as previously discussed, then a histological examination should be performed. It is imperative to acknowledge the paucity of data concerning the impact of delayed diagnosis on survival when routine histology is not performed, as well as the economic implications of the substantial costs associated with routine histological examination. Further studies are required to elucidate this aspect.

## Data Availability

Data are not publicly available due to clinic-internal data protection regulations.
